# Metabolic stasis in an ancient symbiosis: genome-scale metabolic networks from two *Blattabacterium cuenoti* strains, primary endosymbionts of cockroaches

**DOI:** 10.1186/1471-2180-12-S1-S5

**Published:** 2012-01-18

**Authors:** Carmen Maria González-Domenech, Eugeni Belda, Rafael Patiño-Navarrete, Andrés Moya, Juli Peretó, Amparo Latorre

**Affiliations:** 1 Institut Cavanilles de Biodiversitat i Biologia Evolutiva, Universitat de València, P.O. Box 22085, E-46071, València, Spain; 2 Faculty of Pharmacy, University of Granada. Campus of Cartuja, E-18071. Granada, Spain; 3 Departament de Genètica, Universitat de València, Spain; 4 Departament de Bioquímica i Biologia Molecular, Universitat de València, Spain; 5 Centre for Public Health Research (CSISP), E-46020. València, Spain

## Abstract

**Background:**

Cockroaches are terrestrial insects that strikingly eliminate waste nitrogen as ammonia instead of uric acid. *Blattabacterium cuenoti* (Mercier 1906) strains Bge and Pam are the obligate primary endosymbionts of the cockroaches *Blattella germanica* and *Periplaneta americana*, respectively. The genomes of both bacterial endosymbionts have recently been sequenced, making possible a genome-scale constraint-based reconstruction of their metabolic networks. The mathematical expression of a metabolic network and the subsequent quantitative studies of phenotypic features by Flux Balance Analysis (FBA) represent an efficient functional approach to these uncultivable bacteria.

**Results:**

We report the metabolic models of *Blattabacterium* strains Bge (*i*CG238) and Pam (*i*CG230), comprising 296 and 289 biochemical reactions, associated with 238 and 230 genes, and 364 and 358 metabolites, respectively. Both models reflect both the striking similarities and the singularities of these microorganisms. FBA was used to analyze the properties, potential and limits of the models, assuming some environmental constraints such as aerobic conditions and the net production of ammonia from these bacterial systems, as has been experimentally observed. In addition, *in silico* simulations with the *i*CG238 model have enabled a set of carbon and nitrogen sources to be defined, which would also support a viable phenotype in terms of biomass production in the strain Pam, which lacks the first three steps of the tricarboxylic acid cycle. FBA reveals a metabolic condition that renders these enzymatic steps dispensable, thus offering a possible evolutionary explanation for their elimination. We also confirm, by computational simulations, the fragility of the metabolic networks and their host dependence.

**Conclusions:**

The minimized *Blattabacterium* metabolic networks are surprisingly similar in strains Bge and Pam, after 140 million years of evolution of these endosymbionts in separate cockroach lineages. FBA performed on the reconstructed networks from the two bacteria helps to refine the functional analysis of the genomes enabling us to postulate how slightly different host metabolic contexts drove their parallel evolution.

## Background

Recently, the genomes of two different strains of *Blattabacterium cuenoti* (Mercier 1906), Bge and Pam, obligate primary endosymbionts of the cockroaches *Blattella germanica* and *Periplaneta americana*, respectively, have been sequenced [1,2]. *Blattabacterium* constitutes a clade within the class Flavobacteria, the phylum Bacteroidetes, which contains several instances of symbionts of insects, e.g., “*Candidatus* Sulcia muelleri”, obligate endosymbiont of cicadas, spittlebugs and leafhoppers [3], “*Candidatus* Cardinium”, symbiont of the white fly *Bemisia tabaci *[4], and “*Candidatus* Vestibaculum illigatum”, which establishes a symbiosis with the gut flagellate *Staurojoenina sp.* associated to the termite *Neotermes cubanus *[5]. All these endosymbiont bacteria are relatively distant from free-living members within the phylum Bacteroidetes [6]. Thus, if we assume that the age of a symbiotic association of a primary endosymbiont corresponds to the oldest fossil record of its host, we estimate the time of divergence between *B. cuenoti* and its free-living cousins to be 250 Myr [7], thus being possibly one of the most ancient mutualistic insect symbioses described so far.

Cockroaches, natural hosts of *Blattabacterium* sp., excrete waste nitrogen as ammonia [8-11] unlike most terrestrial insects, which eliminate it as uric acid [11]. Ammonotely, the ancestral character present in aquatic animals, has traditionally been considered maladaptive in terrestrial animals [12]. The enigmatic return of cockroaches to ammonotely seems to be related to the role of bacterial endosymbiosis in their nitrogen economy. López-Sánchez *et al. *[1] showed the presence of urease activity in endosymbiont-enriched extracts of the cockroaches *B. germanica* and *P. americana*. Stoichiometric analysis of the core of the reconstructed metabolic networks would suggest that these endosymbiotic bacteria participate in the nitrogen metabolism of the host. Physiological studies ([1,8] and references therein) suggest that uric acid may represent a form of nitrogen storage in cockroaches and that *B. cuenoti* may produce ammonia from uric-derived metabolites provided by the host. In fact, the cockroach fat body contains specialized cells storing uric acid (urocytes) that are in close proximity to the cells containing endosymbionts (bacteriocytes) [13].

A common feature of genomes from bacterial endosymbionts is their strict conservation of gene order and remarkable differential gene losses in the different lineages [14-16]. In the case of the Bge and Pam strains, comparative genomics reveals both a high degree of conservation in their chromosomal architecture and in the gene repertoires (accounting for a total of 627 and 619 genes in Bge and Pam, respectively) despite the low sequence similarity observed (~85% nucleotide sequence identity) [6]. Thus, the metabolic networks of these endosymbionts should be similar, differing only slightly. These differences might be analyzed from a qualitative point of view by comparison between the inferred metabolic maps, but this approach does not allow quantitative evaluation of how these inequalities might affect the functional capabilities of each microorganism. Constraint-based models of metabolic networks represent an efficient framework for a quantitative understanding of microbial physiology [17]. In fact, computational simulations with constraint-based models are approaches that help to predict cellular phenotypes given particular environmental conditions, with a high correspondence between experimental results and predictions [18-20]. It is worth mentioning that they are especially suitable for reconstructed networks from uncultivable microorganism, as it is the case of primary endosymbionts. Thus, Flux Balance Analysis (FBA) is one of these useful techniques for the study of obligate intracellular bacteria, since it reconstructs fluxes through a network requiring neither kinetic parameters nor other detailed information on enzymes [17]. This modeling method is based on the stoichiometric coefficients of each reaction and the assumption of the system at steady-state [21]. FBA calculates metabolites fluxes through the metabolic reactions that optimize an objective function –usually biomass production–, i.e., how much each reaction contributes to the phenotype desired.

In this study, we have reconstructed the metabolic networks of Bge and Pam strains of *B. cuenoti*, focusing on their metabolic abilities and relating them to the symbiotic interaction with their host. Following the protocol proposed by Thiele and Palsson [22], we have quantitatively predicted their biochemical potential by FBA, assuming biomass formation as objective function. In addition, in some simulations we have imposed the constraint of ammonia release from both endosymbionts, in coherence with the physiological observations [8] and as expected by the measured urease activity and the stoichiometric analysis performed by López-Sánchez *et al*. [1]. We have performed sensitivity and robustness analyses and deduced how these endosymbionts may be related to their cockroach hosts metabolically. We offer an overview of the remarkably stable metabolic relationships in these old symbioses as well as providing an explanation for a possible environmental cause of the loss of genes coding for enzymes in a central pathway, such as the TCA cycle in one of the endosymbionts.

## Results

### Metabolic models and FBA simulations

Gene to protein to reaction (GPR) associations were included in the model *i*CG238, corresponding to the reconstructed metabolic network from *B. cuenoti* Bge strain. This model accounted for 238 genes with a known locus in the genome, linked to 296 GPR associations and with 364 associated metabolites. The model *i*CG230 of the reconstructed network of the *B. cuenoti* Pam strain comprised 289 GPR associations, with the participation of 230 genes and 358 metabolites (see Table 1 and Additional Files 1 and 2). Both models included 47 exchange reactions. A difference between the two models deals with the simulated uptake of the sulfur source. Thus, due to the lack of *cys*N, *cys*D and *cys*I genes related to cysteine metabolism in the strain Pam, this model simulates the income of hydrogen sulfide (H_2_S) instead of sulfate, as it is the case in the strain Bge. Although *cys*H and *cys*J genes are present in the genome of the strain Pam, they represent isolated genes within the first steps of the mentioned pathway (see Additional File 3). As a consequence, the following reactions were removed from the final metabolic network: phosphoadenylyl-sulfate reductase (thioredoxin) (EC 1.8.4.8) and sulfite reductase (NADPH) (EC 1.8.1.2), catalyzed by CysH enzyme and by the protein complex CysIJ (CysJ requires the participation of CysI, also missing), respectively.

**Table 1:** Characteristics of metabolic reconstructions from the strains Bge and Pam of *B. cuenoti*.

	Metabolic model	
	
	*i*CG238	*i*CG230
**Protein-encoding genes**	238	230
		
**Metabolites**	364	358
		
Intracellular metabolites	317	311
Extracellular metabolites	47	47
		
**Reactions**	418	411
		
Enzymatic reactions	325	318
Transport fluxes	46	46
Exchange reactions	47	47
		
**Reactions with protein-encoding gene model assignments (GPRs)**	296	289
		
Enzymatic reactions	283	276
Transport fluxes	13	13

**Figure 1:**
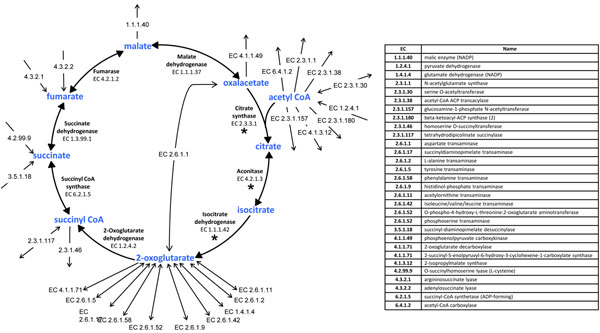
**The TCA cycle and the enzymatic connections of its intermediates.** The only difference between the Bge and the Pam metabolic networks is the absence of citrate synthase, aconitase and isocitrate dehydrogenase in the latter (asterisk labelled steps). Note that, with the exception of their participation in the TCA cycle, citrate and isocitrate are isolated nodes in the network. Each enzymatic step is indicated by its EC number. Double arrows indicate reversible reactions, single arrows indicate irreversible reactions.

Another difference between the Bge and the Pam strain networks is the absence in the latter of the first three steps in the TCA cycle [2]. Missing metabolic steps can generate dead-end metabolites or unconnected nodes in a metabolic network. In other words, an isolated substrate (or product) is generated if it can only be consumed (or produced) by enzymes that are absent in the network [23]. However, we realized that the metabolites leading to citrate (oxaloacetate and acetyl CoA) or the metabolites derived from isocitrate (2-oxoglutarate, coenzymes excluded) are well-connected nodes in both reconstructed networks (Fig. 1), in spite of the absence of the first three steps in the TCA cycle in the strain Pam [2]. On the other hand, both metabolic models showed exactly the same 12 dead-end metabolites (see Additional Files 1 and 2). The reactions leading up to the dead ends were included to obtain a fully functional network. Furthermore, we have considered 75 reactions (33 of them being transport reactions) without any gene associated in either model (Additional Files 1 and 2, and Additional File 4 for further details).

**Figure 2:**
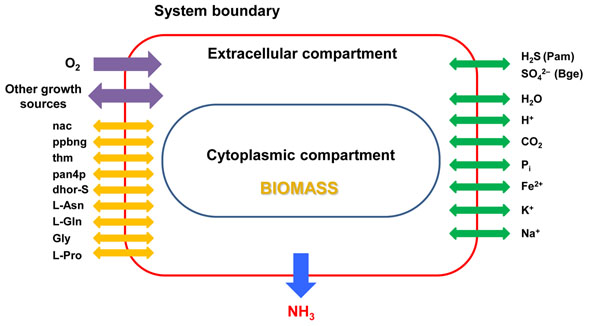
**Metabolite flow in the metabolic models of the endosymbionts.** Metabolites with unconstrained import and export across system boundaries are represented by green arrows (8 metabolites related to usual exchange with extracellular medium) and yellow arrows (9 metabolites supposed to be directly provided by the host). Ammonia is only allowed to leave the system (blue arrow). Other external metabolites (purple arrows) can also alternatively enhance or support (depending on the strain considered) bacterial growth. Abbreviations: nac, nicotinic acid; ppbng, porphobilinogen; thm, thiamin; pan4p, pantotheine-4-phosphate; dhor-S, *S*-dihydroorotate.

**Figure 3:**
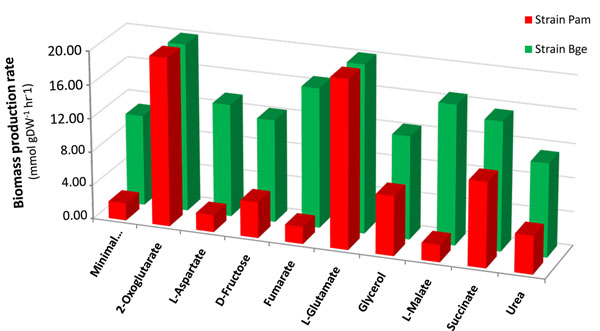
**Effect of different metabolites on the performance of the metabolic models.** Biomass production rates (mmol g DW^-1^ h^-1^) in the two networks (strain Bge, green bars; strain Pam, red bars) were measured under minimal conditions (see Fig. 2 and main text) or considering the uptake of different metabolites.

In order to evaluate the functional phenotype of the metabolic networks from both strains, FBA with biomass production as objective function was employed, using as a reference model the reconstructed network and biomass equation of *E. coli* with some adaptations, as described in Methods. Non-essential amino acids L-Asn, L-Gln, Gly and L-Pro, as well as the compounds (*S*)-dihydroorotate, nicotinic acid, pantotheine-4-phosphate, porphobilinogen and thiamin were supposed to be supplied by the host to meet the biosynthetic needs in both strains, as suggested by the genetic lack of the corresponding synthetic machineries [1,2]. The rest of essential components of the extracellular medium were CO_2_, Fe^2+^, H^+^, H_2_O, K^+^, Na^+^, O_2_, P_i_ and the appropriate sulfur source(Fig. 2). All the above-mentioned chemical components of the environment (host) were necessary and sufficient to yield a viable phenotype in FBA simulations with the *i*CG238 Bge strain model (Fig. 3). However, with the Pam network we obtained a mere 20% of the biomass produced by the Bge network under the same minimal conditions (Fig. 3).

**Figure 4:**
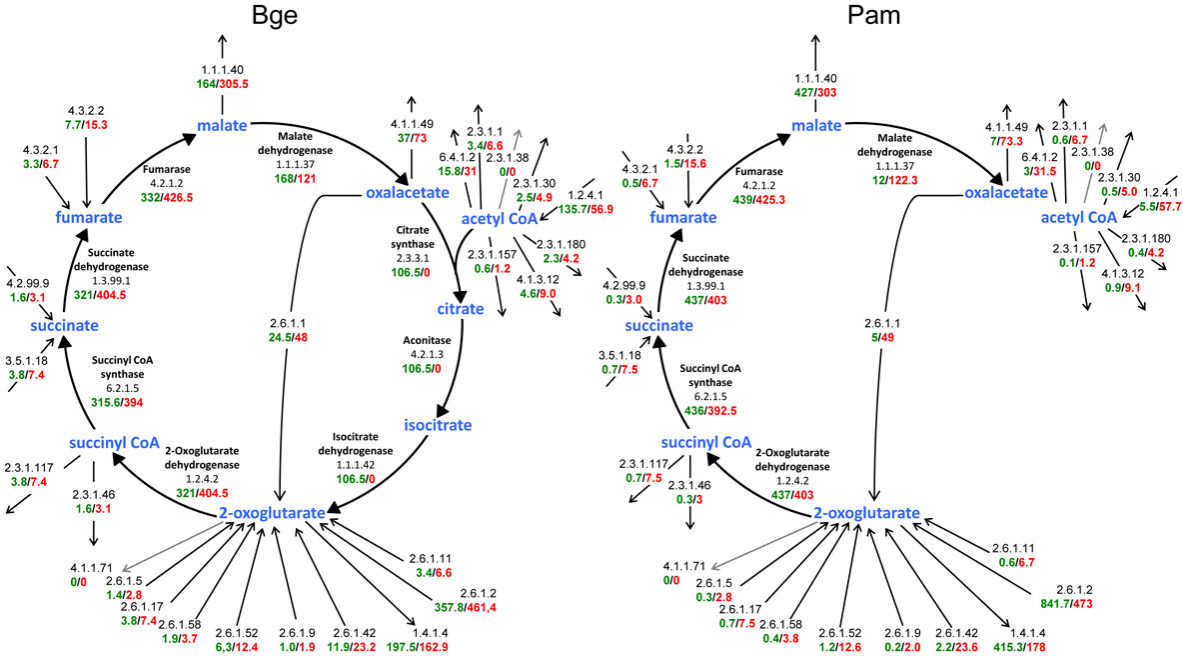
**Flux distribution through the TCA cycle and adjacent reactions.** FBA simulations of both models (strain Bge, left; strain Pam, right) were performed under minimal medium (green values) or with L-Glu uptake (red values). EC numbers are indicated (for enzyme names, see Fig. 1).

FBA was also used to predict the behavior of the strain Bge in terms of growth rate when an additional metabolite was considered in the medium. We tested several metabolites with transport systems encoded by genes present in both *B. cuenoti* genomes (L-Asp, D-fructose, fumarate, L-Glu, glycerol, L-malate, succinate and urea) and also the input of the TCA cycle intermediate 2-oxoglutarate, as a simulation of an anaplerotic reaction. All the considered additions had a positive effect on the biomass production rate by the Bge network, compared to the minimal medium (Fig. 3). In particular, some intermediates of the TCA cycle improved the performance of both networks with a remarkable ten-fold enhancement of biomass production by the Pam network in the presence of L-Glu and 2-oxoglutarate. This result can be correlated with the fact that the strain Pam possesses an incomplete TCA cycle. We decided to focus our attention on how the metabolic flux should be completely redirected through the different reactions leaving or entering this pathway (see Fig. 1). Thus, the fluxes through the transaminase reactions generating 2-oxoglutarate were particularly important because they feed the enzymatic steps of the TCA cycle downstream of the isocitrate dehydrogenase reaction (Fig. 4). In summary, the positive effect of L-Glu (and 2-oxoglutarate) on the Pam network achieved a similar performance to the Bge network due to the anaplerotic effect of these metabolites (Fig. 4).

The excretion of ammonia from the system, a phenomenon compatible with the physiological and experimental observations (for review see [8] and [1] and references therein), was always observed in simulations with both models under minimal conditions. The efflux of ammonia reached maximum levels when L-Glu uptake was simulated by the system. However, the efflux of ammonia stopped and could even be reversed when 2-oxoglutarate or succinate were provided to both metabolic networks. This was due to an increased assimilation of ammonia through displacement of the glutamate dehydrogenase reaction (EC 1.4.1.4) in the assimilative direction.

### Sensitivity and robustness analysis

**Figure 5:**
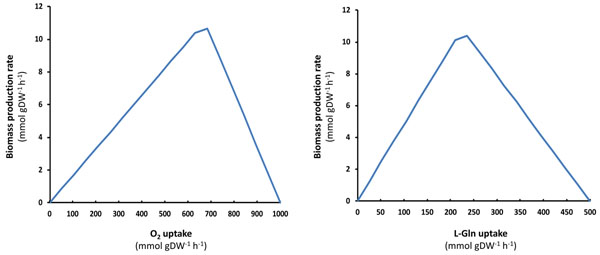
**Effect of oxygen and L-Gln uptake on metabolic network performance.** Biomass production rates (mmol g DW^-1^ h^-1^) by the Bge strain model were measured at different uptake rates of oxygen (left) and L-Gln (right).

The robustness of both metabolic reconstructions, *i*CG238 and *i*CG230, was explored from different perspectives. Firstly, we performed a sensitivity analysis, i.e. how biomass production rate changed as the flux over a specific reaction of interest varied in magnitude. The target reactions to perform this analysis were those involving the exchange of essential and additional growth sources used in the FBA simulations described in the previous section. We also analyzed the effect of oxygen uptake since the metabolic inference from the two cockroach endosymbiont genomes indicates the presence of a complete electron transport chain terminated with a high-affinity *cbb*_3_-type cytochrome oxidase [1,2]. Furthermore, the cockroach fat body, the tissue where endosymbionts are located, exhibits the characteristics of an active aerobic environment (e.g. peroxisome abundance and urate catabolism, [23,1] and references therein). Both the *i*CG238 and the *i*CG230 models, showed a strict dependence on the import of L-Asn, Gly and L-Pro, in accordance with the metabolic inference from the genomes [1,2]. Our simulations using Bge model show that there is a range of metabolic flux values for oxygen and L-Gln exchange reactions over which it is possible to produce an optimum phenotype in terms of biomass (Fig. 5). A similar result was observed for the growth dependence on L-Gln with the Pam model (data not shown).

**Figure 6:**
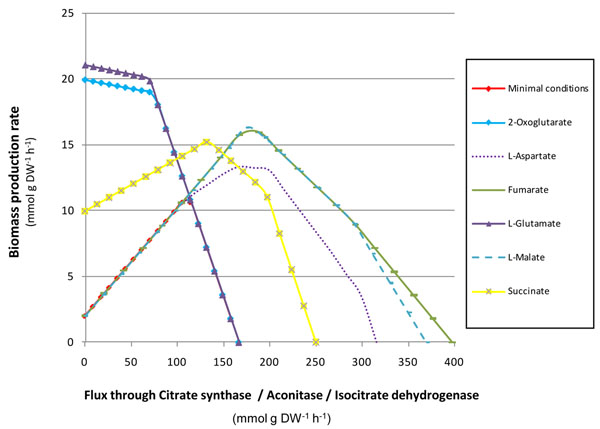
**Sensitivity analysis for the first three reactions of the TCA cycle.** Biomass production rates (mmol g DW^-1^ h^-1^) by the Bge strain model were measured under different metabolic environments (minimal conditions or the uptake of the indicated metabolites, see inset) and diverse reaction flux through the first enzymatic steps of the TCA cycle: citrate synthase, aconitase and isocitrate dehydrogenase.

We also evaluated the sensitivity of the Bge metabolic network to variations in the three first reactions of the TCA cycle, absent in the metabolic network of the strain Pam ([2]; see Fig. 1). We simulated the minimal conditions and those considering the additional uptake of some intermediates of the cycle as well as the anaplerotic amino acids L-Glu and L-Asp, precursors of 2-oxoglutarate and oxalacetate, respectively. As shown in Figure 6, a viable phenotype is produced even when the flux values through the three aforementioned reactions are null. Moreover, the biomass production reaches a maximum value when the flux across such reactions is zero and 2-oxoglutarate or L-Glu is added.

Finally, we also explored the robustness of both metabolic networks by randomly removing genes. The starting point was the aerobic and minimal conditions already described and then we also evaluated the fragility of the network when additional carbon sources were provided to the system. As cutoff value for gene essentiality a >99% decrease in the biomass production after the gene deletion was used, as described by Thomas et al. [24]. For the Bge strain network, a set of essential genes was determined ranging between 76.1 % (minimal medium) and 72.3 % (with added glycerol) of the total genes comprised in the model. With the Pam network we found a genetic essentiality between 79.6 % (minimal medium) and 73.5 % (with added fumarate, L-malate or glycerol).

## Discussion

### Uncultivable bacteria can be studied by *in silico* simulations

In this paper we describe the genome-scale metabolic networks corresponding to two strains of *B. cuenoti*, Bge and Pam, the endosymbiotic bacteria of the cockroaches *B. germanica* and *P. americana*, respectively. Despite the approximately 140-Myr of parallel evolution, both metabolic networks showed striking conservation and we decided to compare their functionality by means of a stoichiometric approach such as FBA. This computational methodology has already been successfully used in a study of the metabolic network robustness of *B. aphidicola*, the primary endosymbiont of aphids, in comparison to *E. coli *[24] and for the simulation of reductive evolution in endosymbionts [25,26]. Thus, FBA represents a valid strategy for the functional study of those bacterial species that pose important obstacles to their empirical study, as it is the case of the uncultivable endosymbionts. In this work we used the *E. coli* model as a reference since to the best of our knowledge there are no empirical data on the biomass function of any members within the phylum Bacteroidetes*.* In the absence of information related to real biomass composition of the modeled organism, the use of the equations of *E. coli* is considered a reliable approach and an acceptable starting point [19,27-29].

The simulations allowed us to identify the minimal environmental components for a functional metabolic network (Fig. 2). For instance, both *Blattabacterium* networks show a strict dependence on L-Gln supply from the host due to the absence of glutamine synthase in both endosymbionts. This dependence of the functionality on the availability of some chemical species may also suggest a possible regulatory role of the external medium in the metabolic behavior of the bacterium. In other biological systems, like the nitrogen-fixing nodules of Leguminosae, oxygen availability modulation by the host has been suggested as a mechanism of punishment to cheaters in the symbiotic relationship [30]. Our *in silico* simulations (Fig. 5) suggest that access to L-Gln and/or oxygen is a good candidate for a control mechanism of cockroaches over their endosymbiotic population.

### Why *Blattabacterium* strain Pam has lost the first three enzymatic steps of the TCA cycle

One of the few differences between the metabolic networks inferred for the Bge and Pam strains of *Blattabacterium* is that the latter has an incomplete TCA cycle (compare [1] and [2]). Sabree and coworkers [6] hypothesized that the two missing enzymes, aconitase (EC 4.2.1.3, *acn*A) and isocitrate dehydrogenase (EC 1.1.1.42, *icd*), in the Pam strain metabolic network, can be functionally substituted by the enzymes 3-isopropylmalate isomerase (EC 4.2.1.33, *leu*C) and 3-isopropylmalate dehydrogenase (EC 1.1.1.85, *leu*B), respectively. However, the first enzymatic step of the TCA cycle (citrate synthase, EC 2.3.3.1, *glt*A) is also absent and apparently there is no other alternative solution to this absent activity. Although the functional substitution of two out of three missing metabolic steps in the TCA cycle cannot be excluded, here we have shown the dispensability of all three genes to obtain a functional phenotype in terms of biomass production under certain conditions. Thus, the proposal of functional substitutions by homologous enzymes is an unnecessary conjecture in this case. There are two reasons: (i) as shown in Figure 1, the lack of the three afore-mentioned steps does not generate true dead-end metabolites, and (ii) there is an alternative way to keep a fully functional metabolic network without the first three enzymes in the TCA cycle.

Our simulations show that the Pam network behaves like the Bge network if an anaplerotic reaction (i.e. the uptake of L-Glu or 2-oxoglutarate) is provided. Under these circumstances, the metabolic fluxes are redirected around the TCA cycle (Fig. 4) and, as shown in Figure 6, the sensitivity analysis demonstrates that the flux through the first three enzymatic steps of the TCA cycle can be null. This behavior may explain the dispensability of the corresponding *glt*A, *acn*A, and *icd* genes if the host provides the endosymbiont with any of the above-mentioned compounds. In other words, the provision of a non-essential amino acid to the endosymbiont by the host may offer a set of biochemical conditions favoring the loss of central metabolic genes in one particular evolutionary lineage.

The loss of these three enzymatic steps in the Pam strain of *Blattabacterium* is an example of how the essentiality of genes may change when the environmental conditions change. Studies of flux connectivity (i.e. reactions that always work together) [31] and synthetic lethality analysis (i.e. searching the effect of multiple gene deletions) [32] have shown that in free-living bacteria, such as *E. coli* or *Helicobacter pylori*, the enzymes coded by the *gltA*, *acnA* and *icd* genes form a subset of essential steps. This enzymatic subset was also determined during our analysis of elementary flux modes in *Blattabacterium* Bge [1]. Thus, it is conceivable that during the transition to intracellular lifestyle, the ancestor of *Blattabacterium* strain Pam found a set of chemical conditions in the host cell making those three formerly essential genes dispensable and thus allowing their loss *en bloc*.

### *Blattabacterium* metabolic networks are fragile

The percentage of essential genes predicted for both *Blattabacterium* strains in gene deletion simulations (ranging from 72.3% to 79.6%) is lower to that determined for *B. aphidicola*, primary endosymbiont of aphids, which showed a fraction of 84% of essential genes in a similar simulation [24]. Those values of genetic essentiality in endosymbiotic metabolic networks are far from the robustness observed in models of free-living bacteria, e.g., around 15% of essential genes coding for metabolic enzymes in *E. coli *[33]. Thus, endosymbiotic metabolic networks are less redundant than networks from free-living bacteria. In comparison to the extreme fragility of a minimalist metabolic network, theoretically deduced from comparative genomics [34] and analyzed by Gabaldón *et al*. [35], with 98% of essential genes, endosymbiont metabolic networks show an intermediate degree of robustness, and may represent different stages of the reductive evolutionary process associated to intracellular lifestyle.

### *Blattabacterium* has a key role in the nitrogen economy of cockroaches

Our working hypothesis is that *Blattabacterium* played a key role during the transition from uricotely to a use of urates as nitrogen storage in cockroaches. The elementary flux mode analysis and the enzymatic assays performed by López-Sánchez *et al. *[1] indicated that the central metabolism of *Blattabacterium* can use urea (and some other nitrogen compounds, as non-essential amino acids) and excrete ammonia. As shown in this work, under minimal conditions the reconstructed metabolic networks of the Bge and Pam strains produce ammonia when biomass growth is optimized. This metabolic performance is compatible with the classical physiological observations made by Cochran and coworkers [8]. In addition, physiological studies with cockroaches indicate that uric acid is a form of nitrogen storage instead of a major waste product like in most insects [8]. According to our hypothesis, the fat body metabolism would produce urea from uric acid and the endosymbiont urease would transform urea into ammonia to be used again, partially by the endosymbiont (i.e. synthesis of Glu via the displacement of the Glu dehydrogenase reaction) and partially by the host, especially for glutamine biosynthesis by Gln synthase. It is remarkable that this enzymatic reaction is absent in *Blattabacterium*, although the metabolic networks of both Bge and Pam strains contain 9 Gln-consuming reactions (in addition to the requirement of Gln for protein synthesis represented by the corresponding tRNA for Gln and a gene coding for glutamine tRNA ligase, *glnS*).

In that context, the retention of a urease in *Blattabacterium* makes evolutionary sense as a key piece of the metabolic mosaic of the cockroach nitrogen economy, whereas the bacterial dependence on a Gln supply by the host contributes to the obligate character of this symbiotic association. The dependence on host-supplied Gln has also been recently described in *Blochmannia vafer*, the primary endosymbiont of the ant *Camponotus vafer*, which contains urease but lacks both Glu dehydrogenase and Gln synthase [36].

The released ammonia observed by physiologists would correspond to the escape of some ammonia produced by the system when all the ammonia-utilizing reactions are saturated, a side effect of the serial transformation from uric acid to urea to ammonia to glutamate/glutamine. In this metabolic framework, our *in silico* modeling was performed with the constraint of ammonia release by the endosymbiont. The mathematical expression of the metabolic networks, thus, helps us understand the systemic properties of the host-endosymbiont relationships. Practically speaking, it serves for the better design of an experimental strategy to functionally characterize the pathway from uric acid to glutamine in cockroaches.

## Conclusions

One of our aims was to perform a genome-scale constraint-based modeling of the metabolisms of two different strains of *B. cuenoti*, Bge and Pam, primary endosymbionts of the cockroaches *B. germanica* and *P. americana*, respectively, which are the result of a parallel evolution during the last 140 million years. A striking feature of the two bacteria is not only the genome architecture conservation, as observed in other similar systems, but also the few gene losses undergone in the different lineages. Thus, both metabolic networks differ from each other in only seven enzymatic reactions. The FBA approach has allowed us to evaluate the different host influences that might explain the loss or retention of certain genes, which is not easy to elucidate *a priori* by visual inspection of the respective metabolic maps. In addition, the fragility shown by the metabolic networks is compatible with a constancy of environmental conditions, and it is the expected outcome for minimal metabolisms derived from the streamlining of endosymbiotic bacterial genomes. The model predictions will allow us to address future functional analyses, and formulate new hypotheses on the metabolic interdependence in the ancient symbiosis between *B. cuenoti* and cockroaches.

## Methods

### Definition of the iCG238 and iCG230 models and FBA simulations

We reconstructed the *i*CG238 and *i*CG230 networks using the *E. coli* K-12 *i*JR904 model as a starting point [37]. From this model, we proceeded as Thomas *et al*. [24] removing all reactions associated with pseudogenes, genes without homologs in those strains or unconnected with the biomass reaction (e.g., *glt*X, *dna*, encoding genes of tRNA ligases and DNA glycosylases). We employed the OrthoMCL algorithm [38] to search for orthologs between *E. coli* K-12 and the different strains of *Blattabacterium* sp. as well as between the two *Blattabacterium* strains in order to obtain a first draft of the metabolic models (inflation thresholds, between 1.2 and 5, choosing in each case the best, normally 1.5 and 3). In addition, the urease reaction, supported by experimental evidence [1], was also added to both models, and ubiquinone was replaced by menaquinone in each metabolic reconstruction since this is the unique or majority quinone in the *Flavobacteriaceae* family [39]. To further curate the models, we performed additional BLAST searches [40] among the corresponding strain of *Blattabacterium*, other flavobacteria and *E. coli* K-12 available in GenBank (e-values below e^-11^), to incorporate reactions either absent in *E. coli* or undetected due to the divergence among strains. In addition, we identified functional domains by means of the interface SMART (Simple Modular Architecture Research Tool) (http://smart.emblheidelberg.de/help/smart_about.shtml) [41,42]. Flux balance analysis (FBA) was performed using the COBRA toolbox [43], a freely available Matlab toolbox and the models were described using the Systems Biology Markup Language (SBML) [44] (Additional Files 5 and 6).

We used the biomass equation derived from the iJR904 *E. coli* model [37] with a few adaptations derived on updated network of such microorganism, i.e. iAF1260 [33]. In particular we added the cofactors thiamine diphosphate and tetrahydrofolate. Additionally, we adjusted the amounts of the four different deoxynucleotide triphosphates in the biomass equation to reflect the GC content of the *Blattabacterium* strains (Bge, 27 mol%; Pam, 28 mol%). Furthermore, since *Blattabacterium* strains are unable to completely synthesize cardiolipin, glycogen, lipopolysaccharide, and spermidine, we removed these components from the biomass equation.

### Robustness analysis

The study of network robustness was performed with the function *robustnessAnalysis* of the COBRA toolbox [43]. In addition, we evaluated the effect of a gene deletion experiment on cellular growth of the resultant mutant using the option *singleGeneDeletion* of the COBRA toolbox. We set to zero the upper and lower flux bounds for the reaction(s) corresponding to the simulated deleted gene. If a single gene is associated with multiple reactions, the deletion of that gene will result in the removal of all associated reactions. On the contrary, a reaction that can be catalyzed by multiple non-interacting gene products will not be removed in a single gene deletion. The possible results of a single deletion are unchanged maximal growth (non-lethal), reduced maximal growth or no growth (lethal). We simulated growth and subsequent fragility analysis with all the different sources which enhance/support biomass formation.

### **List of abbreviations used**

Bge: *Blattabacterium cuenoti* strain of cockroach *Blattella germanica*; DW: Dry Weight; FBA: Flux Balance Analysis; GPR: Gene to protein to reaction association; Pam: *Blattabacterium cuenoti* strain of cockroach *Periplaneta Americana*; TCA cycle: Tricarboxylic acid cycle.

### **Authors' contributions**

CMGD performed the reconstruction process, analyzed the data and evaluated the models, also writing the first draft of the manuscript; EB helped actively in the analyses with COBRA and in drafting the manuscript; RPN helped in the comparative functional analyses between both strains and in drafting the manuscript; AM conceived the study and made important contributions to draft the manuscript; JP conceived and supervised the study and wrote the final manuscript; AL conceived the study and wrote the final manuscript. All authors read an approved the final manuscript.

### **Authors' information**

CMGD: postdoctoral specialist in Microbiology and Systems Biology; EB: postdoctoral specialist in Bioinformatics, Evolutionary Genomics and Systems Biology; RPN: PhD student specialist in Genetics, ‘omics’ Sciences and Bioinformatics; AM: Full Professor of Genetics; JP: Associate Professor of Biochemistry and Molecular Biology; AL: Full Professor of Genetics.

## Competing interests

The authors declare that they have no competing interests.

## Supplementary Material

Additional file 1 Description of the metabolic model of the Bge strain of *B. cuenoti* (host *Blattella germanica*), containing: a list of the GPR associations; a list of the reactions that were supposed to be placed although without any associated gene; a list of the exchange fluxes used in simulations and their constraints; a list of definitions of the metabolite abbreviations; and a list of the dead-end metabolites in the metabolic network. Click here for file

Additional file 2 **Description of the metabolic model of the Pam strain of *B. cuenoti* (host *Periplaneta americana*), containing: the same kind of information as Additional file **1. Click here for file

Additional file 3 **Differences in the cysteine biosynthesis pathway between the strains Bge and Pam.** Sulfate constitutes the sulfur donor in the strain Bge, whereas this function is performed by hydrogen sulfide in the strain Pam. In green, genes exclusively present in *B. cuenoti* (strain Bge); in blue, genes extant in both bacterial strains, Bge and Pam. For all the compounds shown, see the list of abbreviations in the corresponding Metabolites section of Additional files 1 and 2. Click here for file

Additional file 4 Further details on the reconstruction of the networks Click here for file

Additional file 5 Metabolic network model of Bge strain in Systems Biology Markup Language (sbml) format [44], ready to perform simulations with COBRA toolbox [43]. Click here for file

Additional file 6 Metabolic network model of Pam strain in Systems Biology Markup Language (sbml) format [44], ready to perform simulations with COBRA toolbox [43]. Click here for file
