# Supplementary material for: Metabolic stasis in an ancient symbiosis: genome-scale metabolic networks from two Blattabacterium cuenoti strains, primary endosymbionts of cockroaches
Source: BMC Microbiol. 2012 Jan 18;12(Suppl 1):S5. doi: 10.1186/1471-2180-12-S1-S5 (PMC3287516; doi:10.1186/1471-2180-12-S1-S5)
Supplement: Additional file 3 — Differences in the cysteine biosynthesis pathway between the strains Bge and Pam. Sulfate constitutes the sulfur donor in the strain Bge, whereas this function is performed by hydrogen sulfide in the strain Pam. In green, genes exclusively present in B. cuenoti (strain Bge); in blue, genes extant in both bacterial strains, Bge and Pam. For all the compounds shown, see the list of abbreviations in the corresponding Metabolites section of Additional files 1 and 2. [file 1471-2180-12-S1-S5-S3.ppt]

## Slide 1
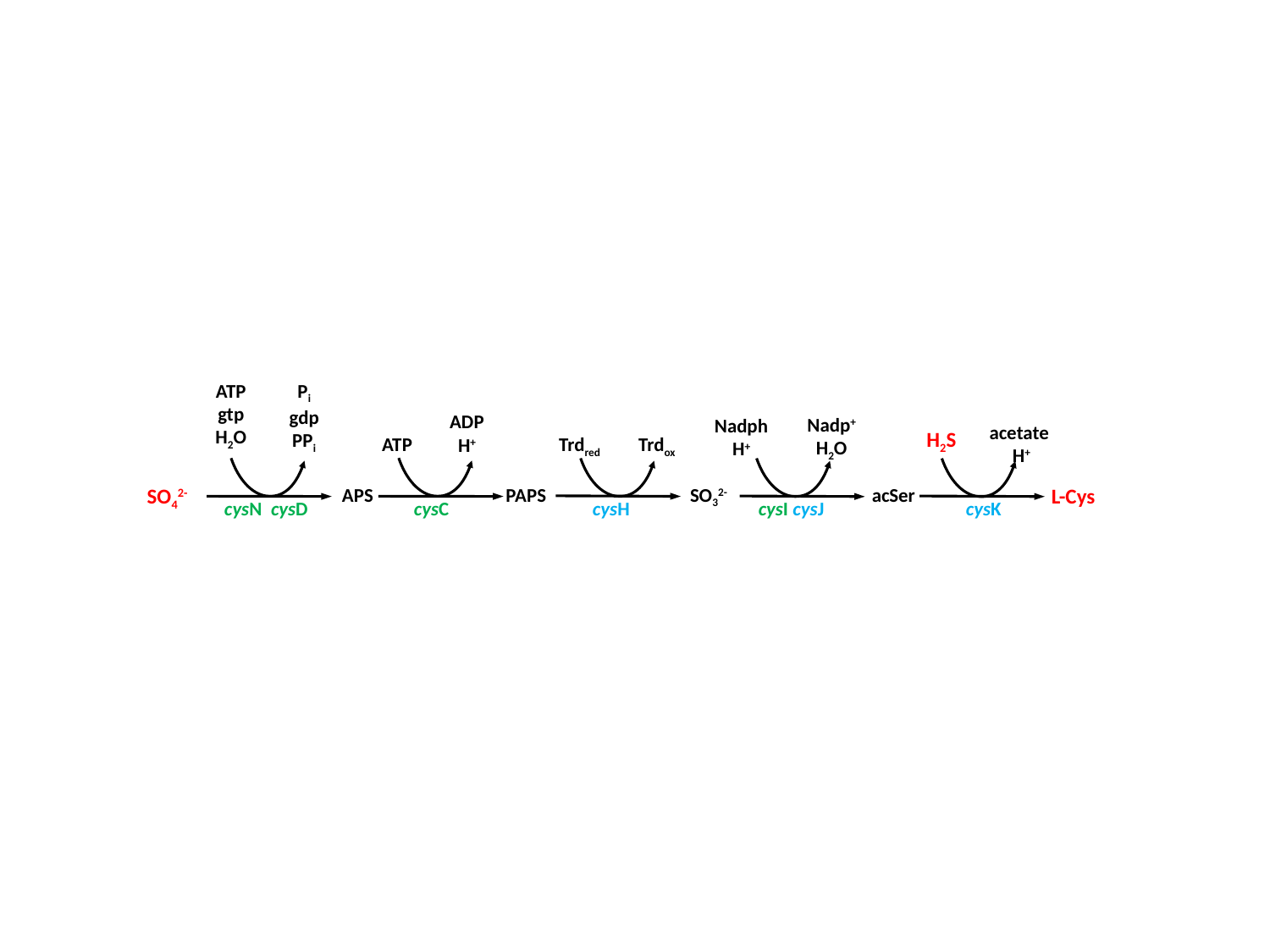

ATP
gtp
H2O
Pi
gdp
PPi
ADP
H+
Nadp+
H2O
Nadph
H+
acetate
H+
H2S
ATP
Trdred
Trdox
SO42-
APS
PAPS
SO32-
acSer
L-Cys
cysN cysD
cysC
cysH
cysI cysJ
cysK
