# Supplementary material for: Metabolic stasis in an ancient symbiosis: genome-scale metabolic networks from two Blattabacterium cuenoti strains, primary endosymbionts of cockroaches
Source: BMC Microbiol. 2012 Jan 18;12(Suppl 1):S5. doi: 10.1186/1471-2180-12-S1-S5 (PMC3287516; doi:10.1186/1471-2180-12-S1-S5)
Supplement: Additional file 4 — Further details on the reconstruction of the networks [file 1471-2180-12-S1-S5-S4.docx]

**Additional file 4. Further details on the reconstruction of the networks**

GPR associations have been manually curated, leading us to refine the annotation of the genomes from the two *Blattabacterium* strains. Furthermore, the gap analysis process of our reconstructions has revealed some previously overlooked genetic data, as well as it has suggested hypotheses for enzymes likely existing in both microorganisms but for which no corresponding gene had been currently annotated. For instance, the analysis of functional domains refined the assigned activities to the ORF corresponding to BLBBGE_512, originally annotated only as *pyr*F (coding for EC 4.1.1.23), whereas we could also detect the functional domain linked to PyrE activity (EC 2.4.2.10). Hence, we propose that this ORF should be *pyr*FE. Regarding the genome of Pam strain, the ORF BPLAN_012 previously associated with the function acetylornithine aminotransferase (EC 2.6.1.11), may also be performing a N-succinyldiaminopimelate aminotransferase activity (EC 2.6.1.17), as its functional domains suggest.

Other instance is the MVA (mevalonate) pathway I. In contrast to most bacteria performing the 1-deoxy-D-xylulose 5-phosphate pathway, both strains of *Blattabacterium* possess all the genes participating in the last steps of the MVA pathway, except for phosphomevalonate kinase (EC 2.7.4.2). However, we have assumed that the second phosphorylation is carried out by some unidentified kinase, perhaps a relaxed version of its homologous enzyme mevalonate kinase (EC 2.7.1.36), encoded by the *mvaK* gene (BLBBGE_085/ BPLAN_549). On the other hand, the ORFs BLBBGE_535 and BLBBGE_283, described as putative septum formation initiator-related protein and phosphoglycerate dehydrogenase (EC 1.1.1.95), respectively, could really be linked to an uridine kinase activity (EC 2.7.1.48, as annotated in the strain Pam) in the case of the former gene, and to an additional erythronate 4-phosphate dehydrogenase activity (EC 1.1.1.290) in the latter ORF, based on sequence homology and functional domain analysis.

In spite of such refinements of both genome annotations and the subsequent definition of new GPRs, we have had to consider 75 reactions not associated to any gene in both models (Table 1 and Additional files 1 and 2). All these reactions are supposed to take place to make possible the flux through both networks. They could be being carried out by not yet defined proteins, by already characterized ones but not following the conventional way of a free-living bacterium or even spontaneously. For instance, the reaction phosphoribosylaminoimidazole carboxylase (EC 4.1.1.21), performed by PurK protein and absent in both strains of *Blattabacterium*, could proceed non-enzymatically since a high concentration of HCO_3_^-^ can partially compensate the defect of the *purK* gene in the absence of purines, as previously observed in *E. coli* mutants [1]. Finally, other network gaps were filled by the addition of transporters like those for the cofactors thiamin and nicotinic acid, as well as the intermediates porphobilinogen, (*S*)-dihydroorotate and pantetheine-4-phosphate, which cannot be synthetized by any of the studied strains of *B. cuenoti* but nonetheless are required for biosynthesis of the heme group, the pyrimidine ring, and coenzyme A, respectively.

**Additional references**

[1] Firestine SM, Poon SW, Mueller EJ, Stubbe J, Davisson VJ: **Reactions catalyzed by 5-aminoimidazole ribonucleotide carboxylases from *Escherichia coli* and *Gallus gallus*: a case for divergent catalytic mechanisms.** *Biochemistry* 1994, **33:**11927-11934.
